# Identification of atypical mitogen-activated protein kinase MAPK4 as a novel regulator in acute lung injury

**DOI:** 10.1186/s13578-020-00484-2

**Published:** 2020-10-19

**Authors:** Ling Mao, Ya Zhou, Longqing Chen, Lin Hu, Shiming Liu, Wen Zheng, Juanjuan Zhao, Mengmeng Guo, Chao Chen, Zhixu He, Lin Xu

**Affiliations:** 1 Special Key Laboratory of Gene Detection & Therapy of Guizhou Province, Zunyi Medical University, Zunyi, 563003 Guizhou China; 2 Department of Immunology, Zunyi Medical University, Zunyi, 563003 Guizhou China; 3 Department of Medical Physics, Zunyi Medical University, Zunyi, 563003 Guizhou China; 4 Department of Laboratory Medicine, Qiannan Medical College for Nationalities, Guizhou, 558000 China; 5 Department of Paediatrics, Affiliated Hospital of Zunyi Medical University, Guizhou, 563000 China; 6 Key Laboratory of Adult Stem Cell Transformation Research, Chinese Academy of Medical Sciences, Guizhou, 563000 China

**Keywords:** MAPK4, ALI, NFKB1, NR3C1, shRNA

## Abstract

**Background:**

Acute lung injury (ALI) is a serious disease with highly morbidity and mortality that causes serious health problems worldwide. Atypical mitogen activated protein kinases (MAPKs) play critical roles in the development of tissues and have been proposed as promising therapeutic targets for various diseases. However, the potential role of atypical MAPKs in ALI remains elusive. In this study, we investigated the role of atypical MAPKs family member MAPK4 in ALI using LPS-induced murine ALI model.

**Results:**

We found that MAPK4 deficiency mice exhibited prolonged survival time after LPS challenge, accompanied by alleviated pathology in lung tissues, decreased levels of pro-inflammatory cytokines and altered composition of immune cells in BALF. Furthermore, the transduction of related signaling pathways, including MK5, AKT, JNK, and p38 MAPK pathways, was reduced obviously in LPS-treated MAPK4^−/−^ mice. Notably, the expression of MAPK4 was up-regulated in lung tissues of ALI model, which was not related with MAPK4 promoter methylation, but negatively orchestrated by transcriptional factors NFKB1 and NR3C1. Further studies have shown that the expression of MAPK4 was also increased in LPS-treated macrophages. Meanwhile, MAPK4 deficiency reduced the expression of related pro-inflammatory cytokines in macrophage in response to LPS treatment. Finally, MAPK4 knockdown using shRNA pre-treatment could ameliorate the pathology of lung tissues and prolong the survival time of mice after LPS challenge.

**Conclusions:**

Collectively, these findings reveal an important biological function of atypical MAPK in mediating the pathology of ALI, indicating that MAPK4 might be a novel potential therapeutic target for ALI treatment.

## Background

Acute lung injury (ALI) and it’s more serious form acute respiratory disease syndrome (ARDS) are critical diseases characterized with diffuse inflammations in lungs, which could be triggered by various pathologies, such as sepsis and severe trauma [1, 2]. Despite numerous therapeutic strategies have been used to ALI treatment [3–5], the worldwide incidence and mortality of ALI are still showing no sign of amelioration in the past decades [6, 7]. One of the reasons why these therapeutic strategies invalid is that the molecule mechanism of development of ALI is very complex and remains to be fully elucidated. Therefore, further investigation on the molecular mechanism of pathology of ALI is still urgent and critical for the development of novel therapeutic strategies against ALI, which ultimately benefits the clinical outcome of ALI patients.

Accumulating evidences have shown that atypical MAPKs play critical roles in the development of tissues and participate in various diseases [8–10]. MAPK4, alias ERK4 or p63 MAPK, is the member of atypical MAPKs and closely related to MAPK6 with 73% amino acid identity in the kinase domain [11]. *Gao *et al. found that miR-127 targeted MAPK4 to activate preadipocyte proliferation [12]. *Menezes-Souza *et al*.* found that MAPK3 and MAPK4 recombinant proteins showed better specificity in the immunodiagnosis of human leishmaniasis than soluble parasite antigen [13]. Most recently, *Wang *et al*.* further reported that MAPK4 overexpression could promote the progression of lung cancer, indicating MAPK4 is a potential target for lung cancer therapy [14]. These findings indicate that MAPK4 play important roles in the development of various types of diseases. However, up to now, the potential role of MAPK4 in the lung related inflammatory diseases including ALI, remains largely unknown.

To this aim, in present study, we first assessed the possible role of MAPK4 in the pathology of ALI using LPS-induced murine ALI model and evaluated the potential value of MAPK4 knockdown on the treatment of ALI. Data showed that MAPK4 deficiency could attenuate the pathology of lung in ALI mice, accompanied by reduced levels of pro-inflammatory cytokines and decreased infiltration of related immune cells, as well as altered transduction of related signaling pathways in lung tissues. Notably, further analysis showed that the expression of MAPK4 was up-regulated in lung tissues of ALI mice, which was not related with MAPK4 promoter methylation, but negatively orchestrated by transcriptional factors NFKB1 and NR3C1. Meanwhile, the expression of MAPK4 was also increased in LPS-treated macrophages, and MAPK4 deficiency could reduce the production of pro-inflammatory cytokines and alter the transduction of related signaling pathways in macrophages. Finally, MAPK4 knockdown could significantly reduce the pathology of lung tissue and prolong the survival time of ALI mice. Altogether, our study reveals an unknown role of MAPK4, a member of atypical MAPKs, in the pathology of ALI, indicating that MAPK4 might be a new therapeutic target for clinic therapy against ALI.

## Results

### MAPK4 deficiency ameliorates the pathology of ALI

**Fig. 1:**
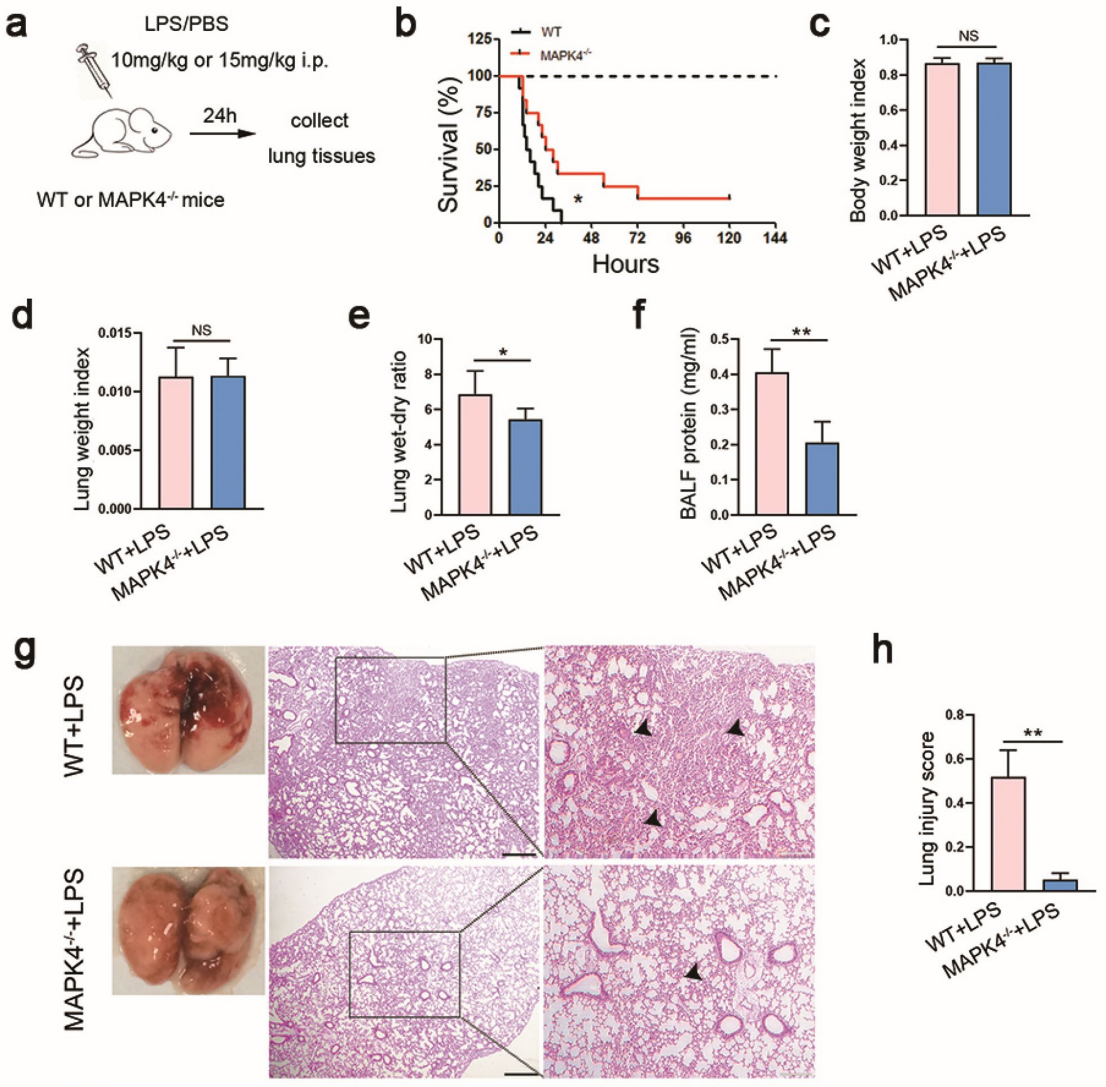
MAPK4 deficiency ameliorates the pathologic injury of ALI. **a** Schematic diagram showing WT C57BL/6 mice or MAPK4^−/−^ mice were treated with i.p. 10 mg/kg or 15 mg/kg LPS, respectively. **b** WT C57BL/6 mice (n = 12) and MAPK4^−/−^ mice (n = 12) were administered with i.p. 15 mg/kg LPS, respectively. The survival time of mice were obtained. WT C57BL/6 mice (n = 5–6) and MAPK4^−/−^ mice (n = 5–6) were administered with i.p. 10 mg/kg LPS, respectively. 24 h later, **c** The weight index, **d** lung weight index (lung weight/weight) and **e** lung wet-dry ratio were obtained, respectively. **f** The protein concentration in BALF was detected. **g** The pathology of lung tissues was analyzed by HE staining. Arrows indicate infiltrated cells. Scale bar = 50 μm. **h** Lung injury score was obtained. Data were presented as the mean ± SEM. *p < 0.05, **p < 0.01

To assess the possible role of MAPK4 in ALI, we utilized MAPK4^−/−^ mice to establish LPS-induced murine ALI model according to our previous work [15]. There was not any significant pathologic change in lung tissues between MAPK4^−/−^ mice and wild type (WT) mice (Additional file 1: Fig. S1a–e), which was consistent with previous study [16]. Importantly, we found that, after LPS treatment, MAPK4^−/−^ mice had a prolonged survival time compared with WT mice (Fig. 1a and b, *P* < 0.05). Even though, the body weight index and lung weight index did not change significantly (Fig. 1c and d, *P* > 0.05), the lung edema, a typical symptom of inflammation in lung injury [17], reduced obviously in LPS-treated MAPK4^−/−^ mice compared with that in LPS-treated WT mice (Fig. 1e, *P* < 0.05). Moreover, the protein level of BALF also decreased markedly in LPS-treated MAPK4^−/−^ mice (Fig. 1f, *P* < 0.05). Furthermore, HE staining results showed that the inflammation in lung tissues reduced significantly in LPS-treated MAPK4^−/−^ mice (Fig. 1g and h, *P* < 0.05). Hence, these data demonstrated that MAPK4 deficiency could obviously alleviate the pathology of LPS-induced ALI.

### Loss of MAPK4 reduces the production of inflammatory cytokines and alters the immune cell composition in ALI

**Fig. 2:**
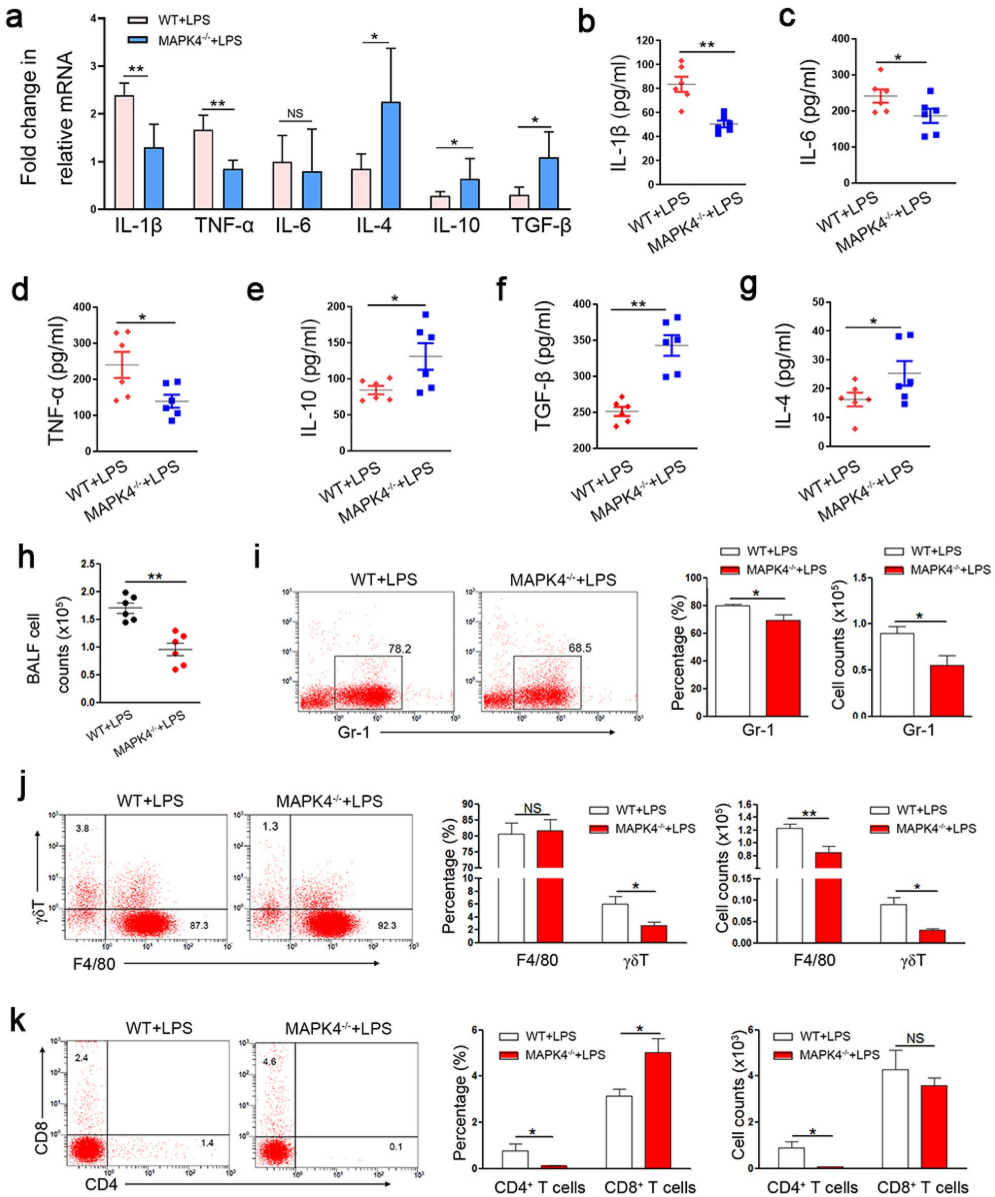
MAPK4 deficiency reduces the production of inflammatory cytokines and alters the composition of immune cells in ALI. WT C57BL/6 mice (n = 5–6) and MAPK4^−/−^ mice (n = 5–6) mice were administered with i.p. 10 mg/kg LPS, 24 h, respectively. **a** The mRNA levels of IL-1β, TNF-α, IL-6, TGF-β, IL-4 and IL-10 in lung tissues were detected by Real-time PCR assay. **b**–**g** The protein levels of IL-1β, TNF-α, IL-6, TGF-β, IL-4 and IL-10 in BALF were detected by ELISA assay. **h** Bronchoalveolar total cell counts were counted. **i** The proportions of Gr-1^+^ neutrophils were analyzed by FCM and the absolute numbers of these cells were calculated, respectively. **j** The proportions of F4/80^+^ Mφ and γδ^+^ T cells were analyzed by FCM and the absolute numbers of these cells were calculated, respectively. **k** The proportions of CD4^+^ T and CD8^+^ T cells were analyzed by FCM and the absolute numbers of these cells were calculated, respectively. Data were presented as the mean ± SEM. *p < 0.05, **p < 0.01

Previous evidences have shown that pro-inflammatory cytokines are involved in the pathology of lung injury [18, 19]. To further monitor the effects of MAPK4 deficiency on the pathology of ALI, we detected the levels of inflammatory related cytokines, such as IL-1β and TNF-α, in ALI mice. Although there was not significant difference in the levels of inflammation related cytokines including IL-1β, IL-6 and TNF-α, as well as IL-4, in lung tissues between MAPK4^−/−^ mice and WT mice (Additional file 1: Fig. S2a-e), the mRNA levels of IL-1β and TNF-α in lung tissues of LPS-treated MAPK4^−/−^ mice significantly decreased compared with those in LPS-treated WT mice (Fig. 2a, *P* < 0.05). By contrast, the mRNA levels of anti-inflammatory cytokines TGF-β, IL-4 and IL-10 were markedly higher in LPS-treated MAPK4^−/−^ mice (Fig. 2a, *P* < 0.05). To verify these data, we also analyzed the protein levels of these cytokines in BALF from LPS-treated WT or MAPK4^−/−^ mice and obtained similar results (Fig. 2b-g, *P* < 0.05). Thus, these results demonstrated that MAPK4 deficiency could reduce lung inflammation by affecting the production of related inflammatory cytokines in ALI.

The infiltration of related immune cells in lung tissues is involved in inflammatory response in ALI [20]. Then, we further assessed the composition of related immune cells in BALF from LPS-treated WT or MAPK4^−/−^ mice. As shown in Fig. 2h, compared with that in LPS-treated WT mice, the total numbers of infiltrated cells in BALF significantly decreased in LPS-treated MAPK4^−/−^ mice (*P* < 0.05). Next, we measured the change on proportion of related immune cells, including Gr-1^+^ neutrophils, F4/80^+^ macrophages, γδT^+^ cells, CD4^+^ T cells and CD8^+^ T cells in BALF from LPS-treated WT or MAPK4^−/−^ mice. Data showed that the proportions and the cell counts of Gr-1^+^ neutrophils, γδT^+^ cells and CD4^+^ T cells obviously decreased in LPS-treated MAPK4^−/−^ mice (Fig. 2i–k,* P* < 0.05). Even though the proportions of F4/80^+^ macrophages did not change significantly, the cell count of F4/80^+^ macrophages still reduced obviously in BALF from LPS-treated MAPK4^−/−^ mice (Fig. 2j, *P* < 0.05). In addition, the cell counts of CD8^+^ T cells did not change significantly (Fig. 2k, *P* < 0.05). Besides, to verify this phenomenon, we also analyzed the possible change on these related immune cells in spleen of WT or MAPK4^−/−^ mice after LPS treatment. Data showed that the cell counts of Gr-1^+^ neutrophils, F4/80^+^ macrophages and γδT^+^ cells also decreased obviously in the spleen of LPS-treated MAPK4^−/−^ mice (Additional file 1: Fig. S3a and b). Combining these results demonstrated that MAPK4 deficiency could affect the infiltration of related immune cells in ALI, which could contribute to the attenuated pathology of ALI.

### MAPK4 deficiency alters the transduction of related signaling pathways in ALI

**Fig. 3:**
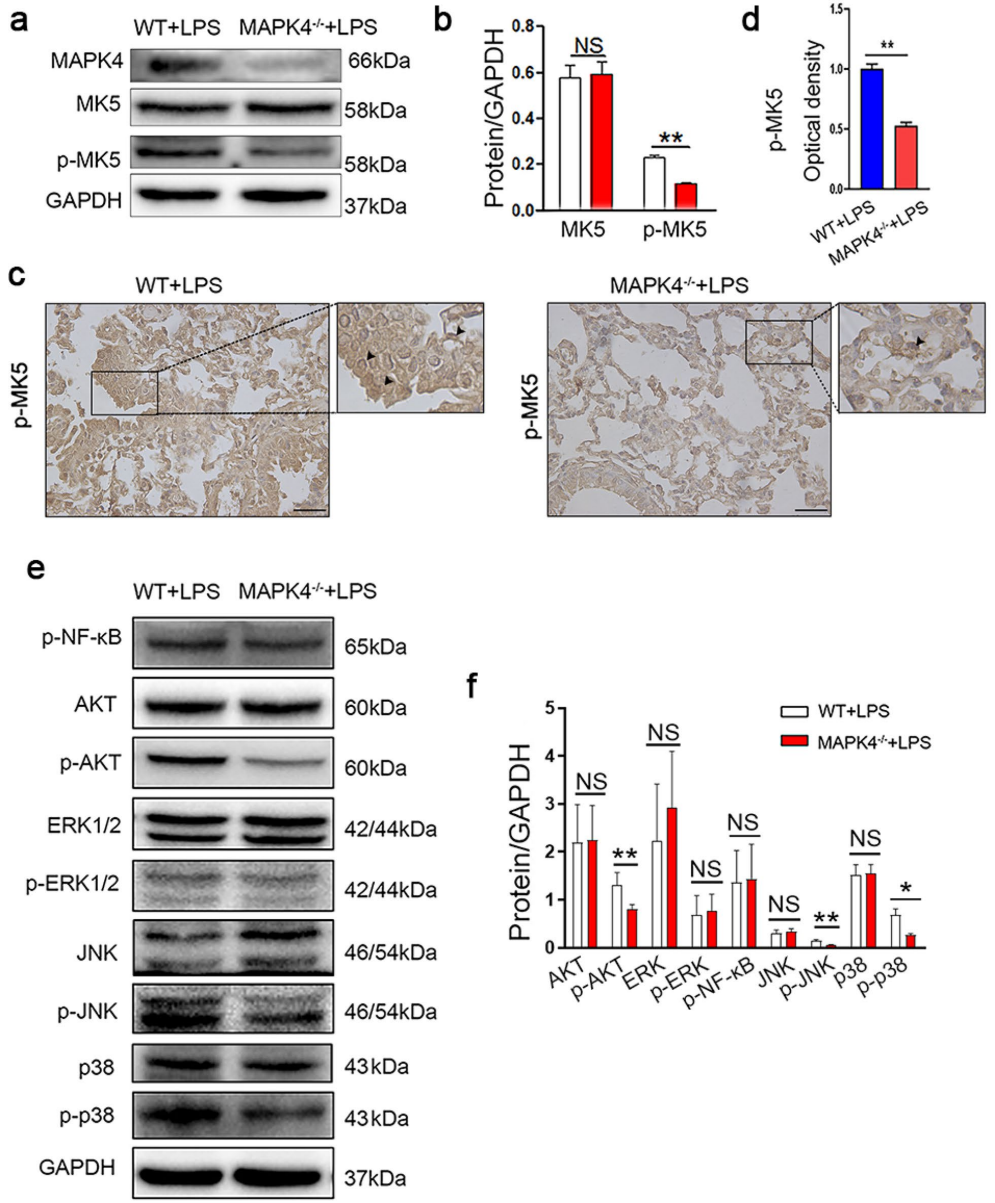
MAPK4 deficiency alters the transduction of related signaling pathways in ALI. WT C57BL/6 mice (n = 5–6) and MAPK4^−/−^ mice (n = 5–6) mice were administered with i.p. 10 mg/kg LPS, respectively. After 24 h, lung tissues were obtained. **a, b** The expressions of MAPK4, MK5 and p-MK5 were detected by Western blot and calculated. **c**, **d** The expressions of p-MK5 were detected by immunohistochemistry and calculated. **e**, **f** The expressions of ERK, p-ERK, AKT, p-AKT, p-NF-κB, p38 MAPK, p-p38 MAPK, JNK and p-JNK were detected by Western blot and calculated. Scale bar = 25 μm. Data were presented as the mean ± SEM. *p < 0.05, **p < 0.01

Next, we investigated the possible effect of MAPK4 deficiency on the transduction of related signaling pathways. Previous study has shown that MK5 is the only reported downstream molecule of MAPK4 [21]. Then, to investigate the possible change of MAPK4 pathway, we detected the expression of MK5 in ALI. Expectedly, the expression of MAPK4 decreased obviously in LPS-treated MAPK4^−/−^ mice (Fig. 3a). Moreover, compared with that in LPS-treated WT mice, the expression of MK5 in LPS-treated MAPK4^−/−^ mice did not change significantly (Fig. 3a and b, *P* > 0.05). Importantly, we found that the level of p-MK5 decreased obviously in LPS-treated MAPK4^−/−^ mice compared with that in LPS-treated WT mice (Fig. 3a and b, *P* < 0.05). To further confirm these data, we also performed immunohistochemistry staining assay and found that the level of p-MK5 markedly reduced in lung tissues in LPS-treated MAPK4^−/−^ mice (Fig. 3c and d, *P* < 0.05), indicating that MAPK4 deficiency could impair the transduction of its downstream pathway.

It is well known that some signaling pathways including AKT, ERK1/2, JNK and p38 MAPK play important roles in the pathology of inflammation response [22, 23]. To reach comprehensive understanding on the role of MAPK4 deficiency on the pathology of ALI, we further detected the possible change on those signaling pathways in lung tissues of LPS-treated WT or MAPK4^−/−^ mice, respectively. Data showed that the levels of AKT, ERK1/2, JNK, p38 MAPK and p-NF-κB did not change significantly in LPS-treated MAPK4^−/−^ mice compared with these in LPS-treated WT mice (Fig. 3e and f, *P* < 0.05). However, the levels of p-AKT, p-JNK and p-p38 MAPK decreased obviously in LPS-treated MAPK4^−/−^ mice (Fig. 3e and f, *P* < 0.05). Taken together, these data indicated that the effects of MAPK4 deficiency on the pathology of ALI was related to the altered transduction of MK5, AKT, JNK and p38 MAPK signaling pathways.

### MAPK4 is up-regulated in the lung tissues of murine ALI model

**Fig. 4:**
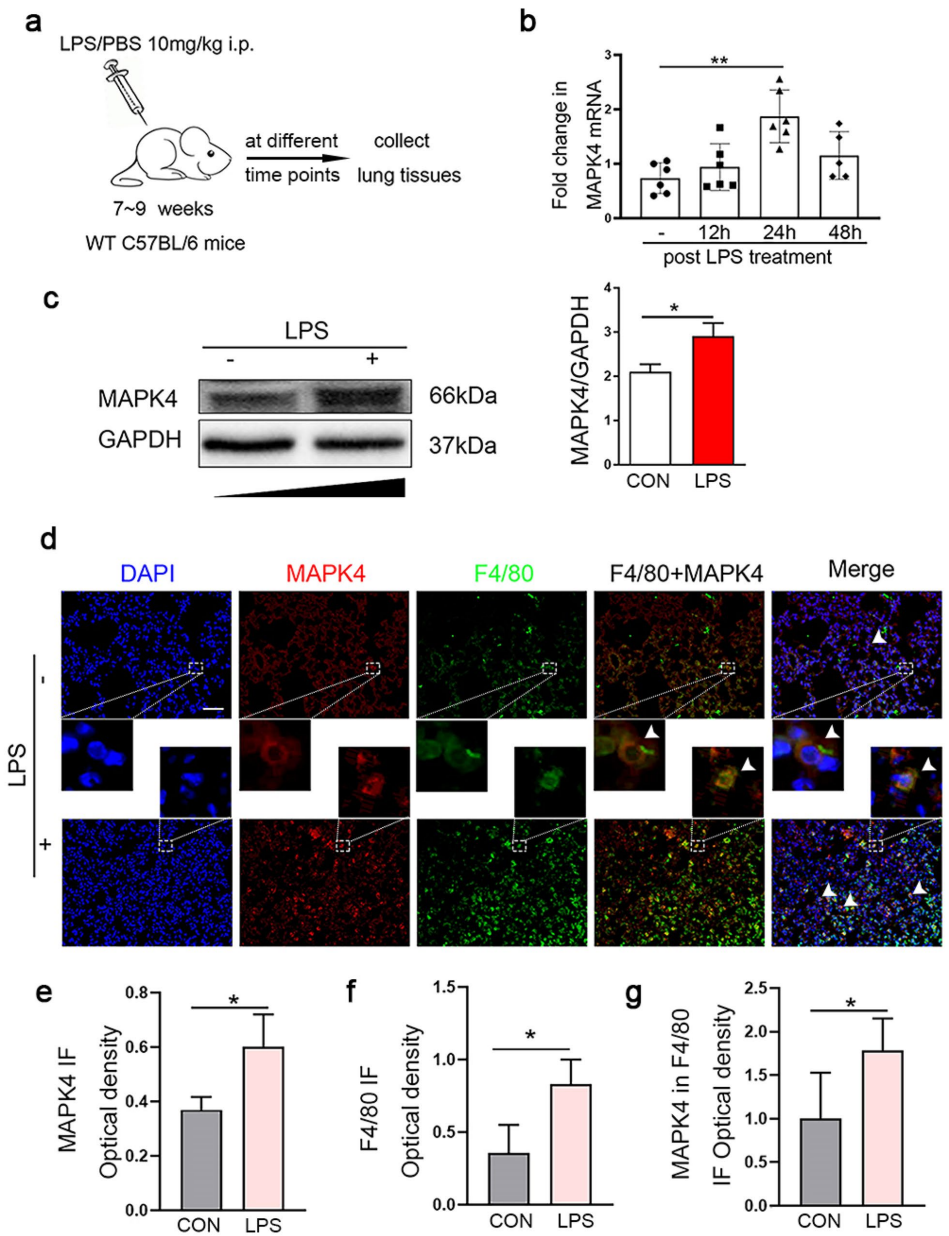
MAPK4 is up-regulated in the lung tissues of murine ALI. **a** WT C57BL/6 mice (n = 5–6) were administered with i.p. 10 mg/kg LPS or PBS. Lung tissues were obtained at different time points. **b** The mRNA levels of MAPK4 in lung tissues were detected by Real-time PCR assay. **c** The protein levels of MAPK4 in lung tissues were analyzed by Western blot and calculated. **d**–**g** The expressions of MAPK4 in F4/80^+^ cells in lung tissues were analyzed by immunofluorescence and calculated, respectively. Arrows indicate F4/80^+^ cells. Scale bar = 50 μm. Data were presented as the mean ± SEM. **p < 0.01

To further investigate the biological role of MAPK4 in ALI, we then detected the expression of MAPK4 in LPS-induced murine ALI model (Fig. 4a). As shown in Fig. 4b, the relative mRNA level of MAPK4 increased obviously in the lung tissues and reached the peak at 24 h post LPS challenge (*P* < 0.05). Moreover, compared with that in control WT group, the protein level of MAPK4 in lung tissues in ALI group also increased significantly (Fig. 4c, *P* < 0.05).

Macrophages, as a dominantly innate immune cell subset expressing TLR4, play important role in the inflammatory process of ALI [24, 25]. Next, to confirm the change of MAPK4 expression in ALI, we also preliminarily assessed the expression of MAPK4 in macrophages in lung tissues of ALI mice. Expectedly, immunofluorescence assay data showed that the infiltration of F4/80^+^ cells increased obviously in the lung tissues in ALI mice compared with control WT mice (Fig. 4d and f, *P* < 0.05). Moreover, the expression of MAPK4 was elevated obviously in lung tissues of ALI mice (Fig. 4d and e, *P* < 0.05), which was consistent with our above data. Importantly, we further found that the expression of MAPK4 dominantly increased obviously in F4/80^+^ cells in lung tissues of ALI mice compared with that in control WT mice (Fig. 4d and g, *P* < 0.05). Altogether, these findings demonstrated that MAPK4 was up-regulated in the lung tissues, at least partially in infiltrated macrophages, of ALI mice.

### NFKB1 and NR3C1 negatively regulate the expression of MAPK4 in ALI

Next, we explore the potential molecular mechanism of up-regulated expression of MAPK4 in ALI. DNA methylation is commonly invoked as a mechanism for transcriptional repression [26–28], we wondered whether DNA demethylation of CpG island in MAPK4 promoter is contributed to the elevated expression of MAPK4 in ALI. Bioinformatics analysis indicated that there was a CpG island in the promoter of MAPK4 (Additional file 1: Fig. S4a). However, massARRAY assay data showed that the CpG methylation level of MAPK4 promoter did not change significantly between control and ALI mice (Additional file 1: Fig. S4b and Fig. S5a, *P* > 0.05), indicating that DNA demethylation of MAPK4 promoter maybe not contribute to the up-regulation of MAPK4 in ALI.

**Fig. 5:**
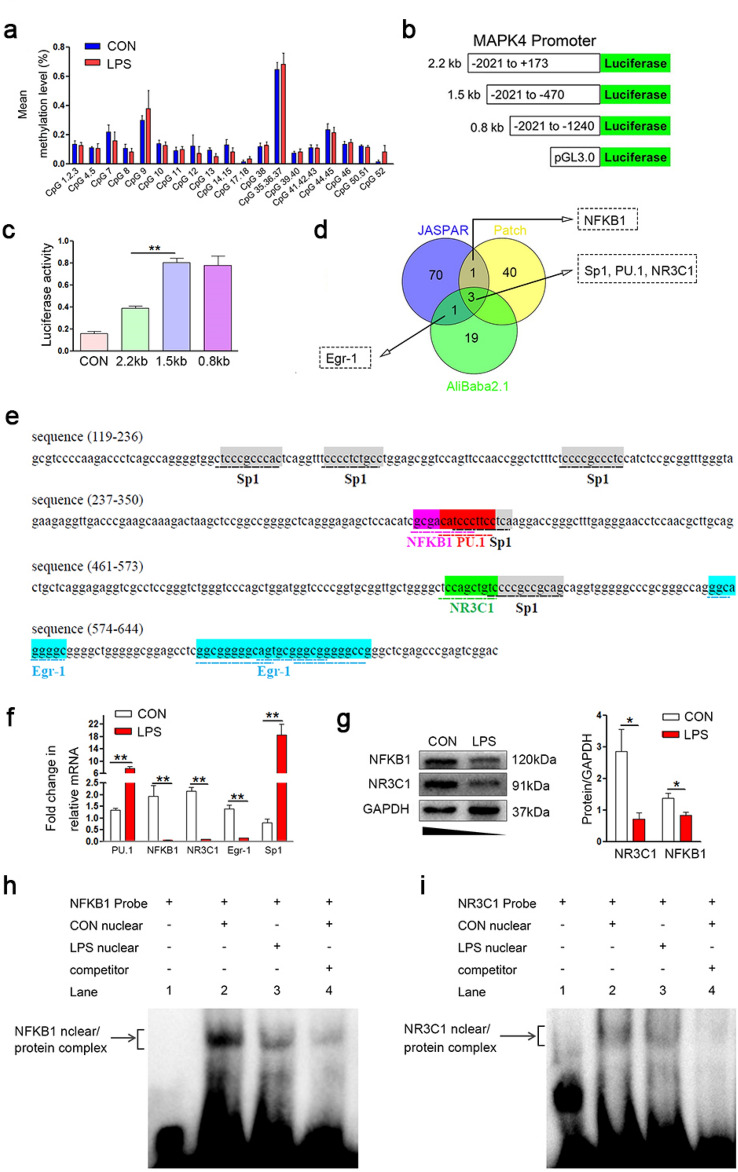
NFKB1 and NR3C1 are the core transcriptional factors of MAPK4 in ALI. **a** Methylation levels of CpG sites in MAPK4 promoter were detected in control lungs (n = 6) and ALI lungs (n = 6). **b** Series truncated versions of MAPK4 promoter (-2021 bp to + 173 bp) were synthesized and cloned into the luciferase reporter vector pGL3.0 basic. **c** The truncated versions of MAPK4 promoter were transfected into RAW264.7 cells and the relative luciferase activity were measured (n = 6). **d**, **e** The candidate transcriptional factors, including Sp1, Egr-1, NFKB1, PU.1 and NR3C1 were screened by TRANSFAC and JASPAP database. **f** The mRNA levels of relative candidate transcriptional factors, including PU.1, NFKB1, NR3C1, Egr-1 and Sp1, were detected in lung tissues by Real-time PCR assay (n = 5–6). **g** The protein levels of NFKB1 and NR3C1 were analyzed in lung tissues by Western blot. **h**, **i** EMSA assay shows DNA–protein interaction of transcriptional factors NFKB1 and NR3C1 to the MAPK4 promoter in vitro. Data were presented as the mean ± SEM. *p < 0.05, **p < 0.01

To further analyze the molecular mechanism of up-regulated MAPK4 in ALI, we next sought to screen the core transcriptional factors of MAPK4 promoter and performed 3′ deletion assay to assess the core sequence of MAPK4 promoter (Fig. 5b). Results showed that the luciferase activity increased significantly between 2.2 and 1.5 kb region in MAPK4 promoter (Fig. 5c, *P* < 0.05), indicating that this region might be the core sequence of MAPK4 promoter. Then, we utilized transcription factor binding sites (TFBS) prediction databases (TRANSFAC and JASPAP) to analyze the potential transcriptional factors and found 5 candidate transcriptional factors according to previous literatures [29–33], including Sp1 (trans-acting transcription factor 1), Egr-1 (early growth response 1), NFKB1 (nuclear factor kappa B subunit 1), PU.1 (spleen focus forming virus proviral integration oncogene) and NR3C1 (nuclear receptor subfamily 3 group C member 1, encoded glucocorticoid receptor) (Fig. 5d and e). Next, we analyzed the mRNA levels of these transcriptional factors and found that, compared with those in control group, the mRNA levels of NFKB1, NR3C1 and Egr-1 were obviously lower in ALI group, while the mRNA levels of Sp1 and PU.1 significantly increased (Fig. 5f, *P* < 0.05). Because the luciferase activity results showed that the putative transcriptional factors could negatively regulate MAPK4 expression and accumulating evidences have shown that NFKB1 and NR3C1 were anti-inflammatory genes [30, 31], so we presumed that NFKB1 and NR3C1 might be putative transcriptional factors in regulating MAPK4 expression in ALI. Expectedly, further analysis showed that the protein levels of NFKB1 and NR3C1 decreased obviously in lung tissues of ALI mice (Fig. 5g, *P* < 0.05), which was consistent with our above data. Importantly, electrophoretic mobility shift assay (EMSA) further showed that NFKB1 and NR3C1 could directly bind to the promoter of MAPK4 (Fig. 5h and i). Together, these results demonstrated that NFKB1 and NR3C1 are new core transcriptional factors in regulating the expression of MAPK4 in ALI.

### MAPK4 deficiency reduces the expression of pro-inflammatory cytokines and transduction of related signaling pathway in macrophages

**Fig. 6:**
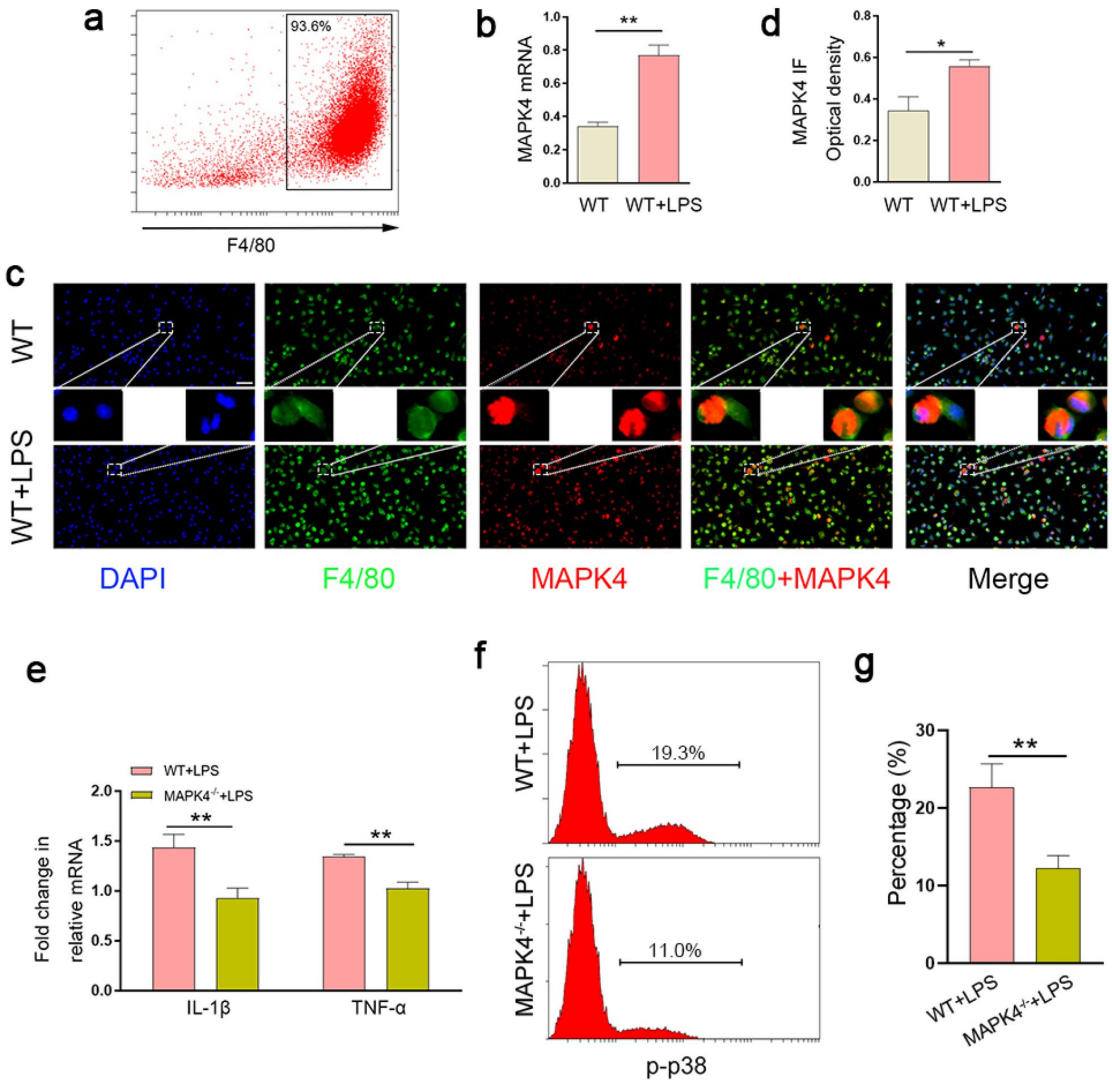
MAPK4 deficiency reduces the expression of inflammatory cytokines and transduction of related signaling pathway in macrophages. **a** Peritoneal macrophages were collected and FCM was used to verify the purity. **b** The mRNA levels of MAPK4 were detected by Real-time PCR assay in WT macrophages and LPS-treated WT macrophages (n = 3). **c**, **d** The protein levels of MAPK4 in WT macrophages and LPS-treated WT macrophages were detected by immunofluorescence and calculated (n = 3). **e** The mRNA levels of IL-1β and TNF-α in LPS-treated WT and MAPK4^−/−^ macrophages were detected by Real-time PCR assay (n = 3). **f**–**g** The expression of p-p38 MAPK was detected by FCM and calculated. Scale bar = 25 μm. Data were presented as the mean ± SEM. *p < 0.05, **p < 0.01

Given the importance of macrophages in pathology of ALI [24, 25] and our above data also showed that macrophage highly expressed MAPK4 in the lungs of ALI mice (Fig. 4d), then, we further investigate the possible role of MAPK4 in macrophages in response to LPS treatment. As shown in Fig. 6a and b, the expression of MAPK4 in macrophages increased significantly after LPS treatment (*P* < 0.05). To confirm this finding, we also performed immunofluorescence assay and obtained similar results (Fig. 6b-d, *P* < 0.05), which is consistent with our above data (Fig. 4d and g). Meanwhile, there was no significant difference on MAPK4 expression in between MAPK4^−/−^ macrophages and LPS-treated MAPK4^−/−^ macrophages (Additional file 1: Fig. S5a-c, *P* > 0.05). Importantly, we analyzed the expressions of inflammatory related cytokines and found that, the expression levels of IL-1β and TNF-α in LPS-treated MAPK4^−/−^ macrophages significantly decreased compared with those in LPS-treated WT macrophages (Fig. 6e, *P *< 0.05). Finally, we further found that, compared with that in LPS-treated WT macrophages, the level of p-p38 MAPK, a representative signaling pathway in our above data, markedly decreased in LPS-treated MAPK4^−/−^ macrophages (Fig. 6f, *P* < 0.05). Combining these data suggested that MAPK4 deficiency could impair the reaction of macrophages in response to LPS treatment.

### MAPK4 knockdown protects mice against LPS-induced ALI

**Fig. 7:**
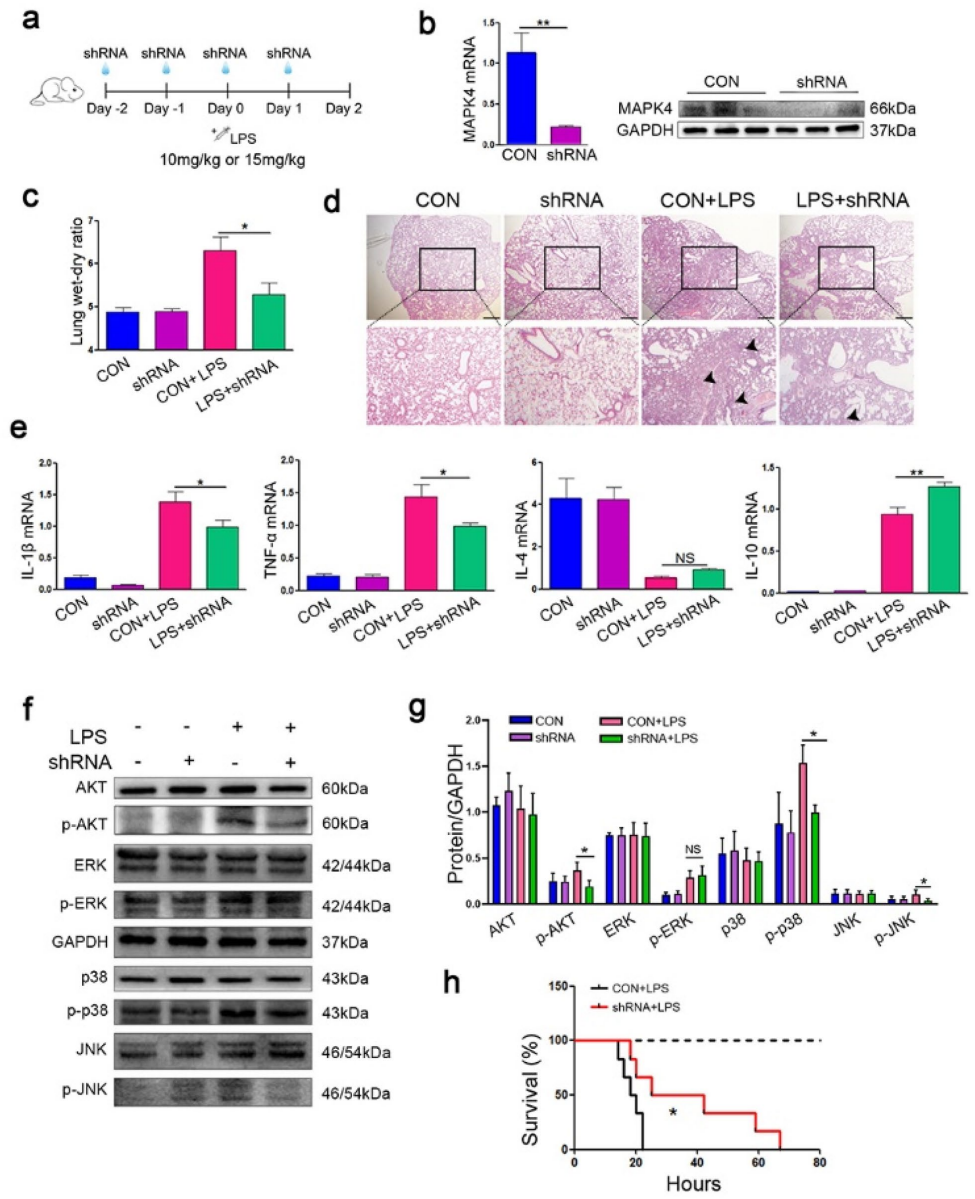
MAPK4-shRNA pre-treatment protects mice against LPS-induced ALI. **a** Schematic diagram showing WT mice were treated with i.t. 10ug MAPK4-shRNA or control vector, then mice were administered with i.p. 10 mg/kg LPS in day 0, respectively. **b** Lung tissues (n = 5–6) were obtained in day 0 and the relative expression of MAPK4 was detected by Real-time PCR assay and Western blot. **c** Lung tissues (n = 5–6) were obtained in day 2 and the lung wet-dry ratio were detected. **d** The pathology of lung tissues was analyzed by HE staining in day 2. Arrows indicate infiltrated cells. Scale bar = 50 μm. **e** The mRNA levels of IL-1β, TNF-α, IL-4 and IL-10 were detected by Real-time PCR assay in day 2. **f**, **g** The expressions of ERK, p-ERK, AKT, p-AKT, p38 MAPK, p-p38 MAPK, JNK and p-JNK were detected and calculated by Western blot in day 2. **h** WT mice (n = 6) were treated with i.t. 10ug MAPK4-shRNA or control vector, then mice were administered with i.p. 15 mg/kg LPS. The survival ratio of mice was recorded, respectively. Data were presented as the mean ± SEM. *p < 0.05, **p < 0.01

Finally, we explored whether MAPK4 might be a novel valuable target in ALI treatment. WT mice were pre-treated with MAPK4-shRNA and then ALI model was induced according to above description (Fig. 7a). As shown in Fig. 7b, compared with that in control group, the expression of MAPK4 decreased markedly in MAPK4-shRNA treatment group (*P* < 0.05). Expectedly, the edema and inflammatory injury of lung tissues reduced obviously in MAPK4-shRNA treatment group (Fig. 7c and 7d). Meanwhile, the levels of pro-inflammatory cytokines, including IL-1β and TNF-α, decreased significantly (Fig. 7e, * P* < 0.05). Conversely, the level of anti-inflammatory cytokine IL-10 increased obviously in MAPK4-shRNA treatment group (Fig. 7e, *P* < 0.05).

To confirm the effect of MAPK4-shRNA on the pathology of ALI, we further analyzed the possible change on the transduction of related signaling pathways. Data showed that the levels of p-AKT, p-JNK and p-p38 MAPK decreased obviously in MAPK4-shRNA treatment mice compared with these in LPS-treated WT mice (Fig. 7f and 7g, *P *< 0.05), which were consistent with our above data. Of note, MAPK4-shRNA treatment could prolong survival time of ALI mice (Fig. 7h). All together, these data demonstrated that MAPK4 knockdown could effectively ameliorate the pathology of ALI, indicating it might be a new valuable therapeutic target in ALI treatment.

## Discussion

Mitogen activated protein kinases (MAPKs) are protein Serine/Threonine kinases that comprise conventional MAPKs (ERK1/2, p38, ERK5, JNK) and atypical MAPKs (ERK3/4, ERK7/8, NLK), which could convert extracellular stimuli into a wide range of cellular responses [34]. Accumulating evidences have demonstrated that conventional MAPKs were involved in the development of ALI [35, 36]. For example, *Carnesecchi *et al*.* found that hyperoxia led to the phosphorylation of JNK and ERK, which was involved in cell death signaling and was related with oxidative stress induced acute lung injury [10]. However, the studies focused on the roles of atypical MAPKs in ALI is still scare. In present study, we found that MAPK4 deficiency could significantly ameliorate the inflammation of lung tissues, accompanied with reduced production of pro-inflammatory cytokines, decreased infiltration of related immune cells and altered transduction of related signaling pathways. Therefore, our current data revealed an unknown role of MAPK4, a member of atypical MAPKs, in the pathology of ALI, which might provide a light on the role of atypical MAPKs in inflammation related diseases.

It is well known that there are complex networks among AKT, JNK and ERK pathways in biological process. For example, *Chen *et al*.* reported that IL-22 tethered to apolipoprotein A-I could ameliorate acute liver injury by altering EKT and AKT signaling transductions [37], suggesting the complicated connection among these pathways in liver injury. Moreover, in our previous work, we also found that miR-7 could affect the development of ALI, accompanied with altered transduction of AKT and ERK pathways [15]. In present study, we found that MAPK4 deficiency could decrease the levels of p-AKT, p-JNK and p-p38 MAPK in ALI model. Similarly, *Wang *et al*.* demonstrated that MAPK4 directly bound and activated AKT by phosphorylation of the activation loop at threonine 308, thereby inducing oncogenic outcomes, including transforming prostate epithelial cells into anchorage-independent growth [14]. However, we didn’t find that the possible influence of MAPK4 deficiency on the expression of p-NF-κB in ALI model, indicating there might be other nuclear regulating pathways. Even though, these data might highlight the underlying connection among conventional MAPKs, atypical MAPKs and other signaling pathways. Previous work found that MAPK4 could regulate MK5 thereby controlling the biological process [21]. Consistent with this finding, we also noticed that, in present study, the level of p-MK5 significantly decreased in the condition of MAPK4 deficiency. Interestingly, *Perander *et al*.* reported that the expression of DUSP2 could inhibit ERK3 and ERK4 mediated activation of its downstream substrate MK5 [38]. Therefore, further study on the roles of other couple molecules of MAPK4, which did not be screened in current study, is much valuable for investigation on the exact connections among MAPK4/MK5, AKT and other signaling pathways in the pathology of ALI.

Recent decades, numerous studies have revealed that the mechanism of transcriptional regulation on gene expression were complex, including DNA methylation and transcriptional factors [39–42]. In present study, we found that the expression of MAPK4 was up-regulated in the lung tissues of ALI mice. Bioinformatics analysis showed that there was a CpG island in the promoter of MAPK4. Unexpectedly, we found that there was not significant change in CpG methylation level of MAPK4 promoter in ALI model, indicating that DNA demethylation of MAPK4 promoter might not be critical for the up-regulation of MAPK4 in ALI. Interestingly, we identified the core sequence region of MAPK4 promoter (-470 to + 173 relative to the TSS). Of note, we verified that two important transcriptional factors NFKB1 and NR3C1 could directly bind to the core sequence region of MAPK4 promoter, indicating that NFKB1/NR3C1 negatively regulated the expression of MAPK4 in ALI. Consistent with these findings, *Adamzik *et al*.* showed that genotypes of NFKB1 promoter polymorphism were associated with the severity in ARDS [43]. Moreover, *Zhao *et al*.* reported that somatostatin could alleviate the pathology of murine ALI, which was closely related with the affinity of glucocorticoid receptor [44]. Therefore, these data further verified the important roles of NFKB1 and NR3C1 in the pathology of ALI.

The gene silencing technique, such as antisense oligonucleotides (ASOs) and short hairpin RNA (shRNA), could effectively knock down the expressions of interesting genes and is an important strategy for gene therapy against various diseases [45, 46]. For instance, Wei et al. reported that MALAT1-shRNA treatment alleviated the inflammatory injury after lung transplant ischemia–reperfusion by downregulating IL-8 and inhibiting infiltration and activation of neutrophils [47]. Similarly, our previous work also showed that ASOs against miR-21 could reduce the growth and metastasis of human CRC cells in vivo and in vitro [48]. To evaluate the potential value of MAPK4 targeting in ALI treatment, herein, we assessed the possible effects of MAPK4-shRNA pre-treatment on the pathology of ALI. Expectedly, we found that MAPK4 knockdown using shRNA pre-treatment could obviously reduce the pathology of lung tissues in ALI model, accompanied by altered levels of inflammatory cytokines and transduction of related signaling pathways. Importantly, we noticed that MAPK4 knockdown could prolong the survival time of ALI mice. Combining these data, we demonstrated that MAPK4 might be a new valuable therapeutic target for ALI therapy. Finally, it would be interesting that, in present study, we noticed that macrophages dominantly expressed MAPK4 in lung tissues of ALI. Furthermore, the expression of MAPK4 increased obviously in LPS-treated macrophages. Importantly, MAPK4 deficiency could significantly impair the expression of pro-inflammatory cytokines and transduction of related signaling pathways in macrophages in response to LPS treatment. Even though the potential roles of MAPK4 in other immune cells such as neutrophils and T cells remain largely unknown, given the fact that the important role of interactions among macrophages and other immune cells, such as neutrophils and T cells, in the pathology of ALI, therefore, successive works on the potential roles of MAPK4 in the biological function of these immune cells might be much valuable for the illustration of the role of MAPK4 in ALI and will be helpful for the development of MAPK4-based targeting therapy for ALI.

## Conclusions

In all, for the first time, our study revealed an unknown role of atypical MAPKs member MAPK4 in the pathology of ALI. Furthermore, we found that the expression of MAPK4 was up-regulated in ALI, which was negatively orchestrated by transcriptional factors NFKB1 and NR3C1. Importantly, MAPK4 knockdown could obviously reduce the pathology of lung tissue and prolong survival time of ALI mice. Therefore, the current work might not only provide a novel insight on the biological role of atypical MAPKs in the pathology of ALI but also will be helpful for the development of novel therapeutic target for ALI treatment.

## Materials and methods

### Mice

MAPK4 deficiency (MAPK4^−/−^) mice breeding pair in a C57BL/6 background were purchased from The Jackson Laboratory (USA, 027666). Animals were housed under specific pathogen-free conditions at Zunyi Medical University

### Cell culture

Raw264.7 cells were purchased from Conservation Genetics CAS Kunming Cell Bank (China, KCB200603YJ), and were cultured in high glucose DEME containing 10% fetal bovine serum at 37 °C in 5% CO_2_.

### Peritoneal macrophage preparation and stimulation

WT and MAPK4^−/−^ mice (7 to 9 week-old) were sacrificed, and the peritoneal cavity was lavaged with cold PBS. The peritoneal cells were collected by centrifugation and seeded in the cell culture plate. Macrophages were allowed to adhere for 6 h, washed with fresh medium to remove unattached cells, and incubated overnight. For the stimulation experiments, macrophages were stimulated with LPS (100 ng/mL) for 24 h, then the cells were collected for the further analysis.

### Establishment of ALI Model

WT and MAPK4^−/−^ mice (7 to 9 week-old) were challenged with i.p. injection of 10 mg/kg LPS (Sigma, USA, Escherichia coli 0111:B4) dissolved in sterile PBS as shown in our previous study [15]. Then the body weight and lung weight index (lung weight/body weight) was detected at indicated time.

### Bronchoalveolar Lavage

Immediately after euthanasia, 1 ml aliquots of PBS were slowly infused in the murine lungs through the tracheostomy and then withdrawn gently. This lavage was repeated three times using the same syringe. The collected lavage fluid was stored in a 10 ml tube on ice. The fluid was centrifuged at 1000 rpm and 4 °C for 10 min, and the cell sediment was washed with PBS. The cell-free supernatant was centrifuged again at 14,000 g and 4 °C for 10 min, stored at − 80 °C and used for determination of cytokines content via ELISA. To the pellet, red blood lysis buffer (Solarbio, China, R1010) were used for 15 min and washed with PBS. Next, the pellet was resuspended for analysis.

### Lung edema determination

Lungs from mice were excised and completely dried in the oven at 60 °C 24 h for calculation of lung wet/dry ratio.

### Histology and immunohistochemistry

Lung tissues were fixed in 4% paraformaldehyde, embedded in paraffin, and cut into 4 μm-thick sections. For histology analysis, the lung sections were stained with hematoxylin and eosin (HE). For immunohistochemistry, the lung sections were deparaffinized with xylene and rehydrated in graded ethanol (100% to 70%). To eliminate endogenous peroxidase activity, the slides were treated with 3% H_2_O_2_ for 30 min and washed with PBS. Then, the slides were blocked with Goat serum (BOSTER, China, AR1009) for 2 h at room temperature and incubated overnight at 4 °C with corresponding antibody (p-MK5: Biorbyt, UK, orb5579). After washed with PBS, the slides were incubated with secondary antibodies and visualized with DAB kit (Solarbio, China, DA1010). Finally, the slides were counterstained with hematoxylin and analyzed by Olympus microscope. The histopathology of the lung injury was scored quantitatively as previously described [49]. Briefly, five random fields of five dimensions of histology features were evaluated by 5 parts: (A) neutrophils in the alveolar space, (B) neutrophils in the interstitial space, (C) hyaline membranes, (D) proteinaceous debris filling the airspaces, and (E) alveolar septal thickening.

### Immunofluorescence

Lung tissues were fixed in 4% paraformaldehyde, embedded in paraffin, and cut into 4 μm-thick sections. Briefly, the slides were incubated with corresponding primary antibodies (MAPK4: Proteintech, USA, 26102–1-AP; F4/80: Abcam, UK, ab60343) and secondary antibody (Alexa 647: Cell Signaling Technology, USA, 4414S) after deparaffinization and rehydration. Then, the slides were counterstained with DAPI (Beyotime, China, C1002) and observed by Olympus microscope.

### RNA extraction and quantitative real time PCR

Total RNA was isolated from mice lungs using RNAiso Plus (TAKARA, Japan, 9108) according to manufacture’s instructions. RNA was quantified and reverse-transcribed according to manufacture’s instructions (TAKARA, Japan, RR037A). SYBR Green-based real time quantitative PCR reactions (TAKARA, Japan, RR820A) and gene specific primers were used. The following primers were used: IL-1β forward: 5′-TGCCACCTTTTGACAGTGATG-3′, reverse: 5′-AAGGTCCACGGGAAAGACAC-3′; IL-6 forward: 5′-GGAAATCGTGGAAATGAG-3′, reverse: 5′-AGGACTCTGGCTTTGTCT-3′; TNF-α forward: 5′-CAGGGGCCACCACGCTCTT C-3′, reverse: 5′-TTTGTGAGTGTGAGGGTCTGG-3′; IL-4 forward: 5′-AACGAGGTCACAGG AGAA-3′, reverse: 5′-CCTTGGAAGCCCTACAGA-3′; IL-10 forward: 5′-TACAGCCGGGAAG ACAATAA-3′, reverse: 5′-AGGAGTCGGTTAGCAGTATG-3′; TGF-β forward: 5′-GGCGGTGC TCGCTTTGTA-3′, reverse: 5′-TCCCGAATGTCTGACGTATTGA-3′; MAPK4 forward: 5′-CCAAAGCATCCCTCAGTTGT-3′, reverse: 5′-CAAGGGGTTGGAAGTCAATG-3′; GAPDH forward: 5′-TCCATGACAACTTTGGCATTG-3′, reverse: 5′-TCACGCCACAGCTTTCCA-3′. Gene expression levels were quantified using Bio-Rad CFX96 detection system (Bio-Rad, USA). With GAPDH was used as internal reference, the expressions of genes were calculated by using the comparative threshold cycle (Ct) method.

### DNA extraction and methylation analysis

Genomic DNA was extracted from 12 lungs of control and ALI model mice by using DNeasy Blood and Tissue kit (QIAGEN, Germany, 69504) according to manufacture’s instructions. The quality and quantified were evaluated by gel electrophoresis and a NanoDrop spectrophotometer (Thermo, USA). The genomic DNA from each sample was treated with sodium bisulfite using an EZ DNA methylation kit (Zymo Reasearch, USA). The MassARRAY platform (The Beijing Genomics Institute, China) was used for quantitative analysis of MAPK4 methylation. We used the primers (5′-aggaagagagGGGTGGGTTTTATTAGAGATAGTGG-3′, 5′-cagtaatacgactcactatag-ggagaaggctAATCTAAATCCCAACTAAATAATCCC-3′) to amplify the region of each promoter. Altogether, 35 CpG sites were tested in this region. The spectra methylation ratios of each CpG site were generated by MassARRAY EpiTYPER software (Agena, USA).

### FCM

Surface markers of series immune cells were detected by flow cytometry (FCM) with Beckman Gallios (Beckman Coulter, USA). FCM was performed on Beckman Gallios with CellQuest Pro software using directly anti-Mouse monoclonal conjugated antibodies against the following markers: F4/80-Percp-Cy5.5 (no.45-4801-82), γδT-APC (no.17-5711-81), NK1.1-APC (no.17-5941-81), CD11c-PE (no.12-0114-82), CD4-PE-Cyanine7 (no.25-0041-82), CD8-Percp-Cy5.5 (no.45-0081-82), CD62L-PE (no.12-0621-81), CD69-APC (no.17-0691-82), Gr-1-PE-Cyanine7 (no.25–5931-82), CD86-APC (no.17-0682-81), MHCII-PE (no.12-5321-81), with corresponding isotype-matched (Thermo Fisher, USA). Cells were stained with corresponding antibodies (1:100) at 4 °C for 30 min, respectively. After washing twice, stained cells were analyzed with a Beckman coulter flow cytometer.

### ELISA

The protein levels of IL-1β, TNF-α, IL-6, IL-4, IL-10 and TGF-β in BAL fluid were detected by ELISA according to manufacture’s instructions, respectively (Thermo Fisher, USA).

### Western blot

Lung tissues were homogenized in ice-clod lysis buffer (KeyGEN BioTECH, China, KGP2100) according to manufacture’s instructions. Equal amounts of protein were separated by 10% SDS-PAGE and protein were transferred onto polyvinyldifluoride membranes. Membranes were incubated with 5% skim milk in PBS for 1 h. Immunoblotting was performed using mAbs to MAPK4 (Abcam, UK, ab96816), AKT (Cell Signaling Technology, USA, 4691), p-AKT (Cell Signaling Technology, USA, 4060), ERK1/2 (Cell Signaling Technology, USA, 4695), p-ERK1/2 (Cell Signaling Technology, USA, 4370), p-NF-κB (Cell Signaling Technology, USA, 3039S), p-JNK (Cell Signaling Technology, USA, 4668), p-p38 MAPK (Cell Signaling Technology, USA, 4511S), MK5 (Cell Signaling Technology, USA, 7419S), p-MK5 (Biorbyt, UK, orb5579) and GAPDH (Cell Signaling Technology, UK, 5174). Membranes were washed in PBST and subsequently incubated with a secondary anti-rabbit antibody conjugated to HRP (Cell Signaling Technology, USA, 7074S). The signal was detected and analyzed using Bio-Rad ChemiDoc MP Imaging System (Bio-Rad, USA). GAPDH was used as internal reference.

### Plasmid construction

Series versions of truncated MAPK4 promoter (NCBI, NC_000084.6 74064925 to 74067228) were synthesized and cloned into pGL3.0 basic vector between KpnI and MluI sites (Gene Create, China, GS1-1905109). And we synthesized MAPK4-shRNA (forward: 5′-GATCCGCAAGGGTTATCTGTCAGAAGGGTTGTTCAAGAGACAACCCTTCTGACAGATAACCCTTGTTTTTTACGCGTG-3′, reverse: 5′-AATTCACGCGTAAAAAACAAGGGTTATCTGTCAGAAGGGTTGTC TCTTGAACAACCCTTCTGACAGATAACCCTTGCG-3′) and control group (forward:5′-GATCCGCAAATTGGTCTGACTGGAAGGGTTGTTCAAGAGACAACCCTTCCAGTCAGACCAATTTGTTTTTTACGCGTG-3′, reverse: 5′-AATTCACGCGTAAAAAACAAATTGGTCTGACTG GAAGGGTTGTCTCTTGAACAACCCTTCCAGTCAGACCAATTTGCG-3′), then cloned into pLVX-shRNA1 vector between BamHI and EcoRI sites. These vectors were extracted by using EndoFree Plasmid Maxi Kit (QIAGEN, Germany, 12,123). After verified by DNA sequencing, these vectors were used for further study.

### Transfection and luciferase reporter assay

Raw264.7 cells were transfected with series of truncated MAPK4 promoter vectors by using Lipofectamine 3000 reagent (Invitrogen, USA, L3000015) according to manufacture’s instruction. After 24 h, the cells were detected for Luciferase activity according to the manufacture’s instruction (Promega, USA).

### shRNA treatment

At 2 days prior to LPS, MAPK4-shRNA was used to inhibit MAPK4 expression, while a control shRNA was used as a control group. A 10ug vector with transfection reagent (Engreen, UK, 18668-11-2) was dripped into nasal cavity. After 2 days treatment with MAPK4-shRNA or MAPK4-control, mice were challenged with 10 mg/kg LPS by intraperitoneal injection. Then, the following 2 days, mice were treated with MAPK4-shRNA or MAPK4-control. Finally, the lung tissues were collected in day 5.

### EMSA

Nuclear proteins were extracted from the lung tissues of control or ALI mice by using Nuclear and Cytoplasmic Extraction Regents (Thermo Fisher, USA, 78,833) according to manufacture’s instruction. The biotinylated and un-biotinylated probes (NFKB1: forward:5′-GAGCTCCACATCGCGACATCCCTTCCTCAAGGAC-3′, reverse: 5′- CTCGAGGTGTAGCGCTGTAGGGAAGGAGTTCCTG-3′; NR3C1: forward:5′-GTTGCTGGGGCTCCAGCTGTCCCCGCCGCAGCA-3′, reverse: 5′-CAACGACCCCGAGGTCGACAGGGGCGGCGTCGT-3′) were synthesized (Sangon, China). The electrophoretic mobility shift assays for each transcription factor were performed using Chemiluminescent Nucleic Acid Detection Module Kit (Thermo Fisher, USA, 89880) according to manufacture’s instruction.

### Statistical analysis

Statistical analysis was performed using GraphPad Prism 5 software. 1-way ANOVA followed by Bonferroni’s post-hoc was applied for multiple comparisons and student’s t-test was used when two conditions were compared. *P* < 0.05 was considered statistically significant and two-sided tests were performed. All data are shown as a mean ± standard error of the mean (SEM). Survival was evaluated by the Kaplan–Meier method.

## Supplementary information

**Additional file 1.** Additional figures.

## Data Availability

The datasets generated and analyzed during the current study are available from the corresponding author on reasonable request.

## Abbreviations

MAPK4: Mitogen activated protein kinases 4
ALI: Acute lung injury
NFKB1: Nuclear factor kappa B subunit 1
NR3C1: Nuclear receptor subfamily 3 group C member 1
shRNA: Short hairpin RNA
BALF: Bronchoalveolar lavage fluid
