# Supplementary material for: Identification of atypical mitogen-activated protein kinase MAPK4 as a novel regulator in acute lung injury
Source: Cell Biosci. 2020 Oct 19;10:121. doi: 10.1186/s13578-020-00484-2 (PMC7570399; doi:10.1186/s13578-020-00484-2)
Supplement: Supplementary file 1 — Additional file 1. Additional figures. [file 13578_2020_484_MOESM1_ESM.docx]

**Additional figure**

**
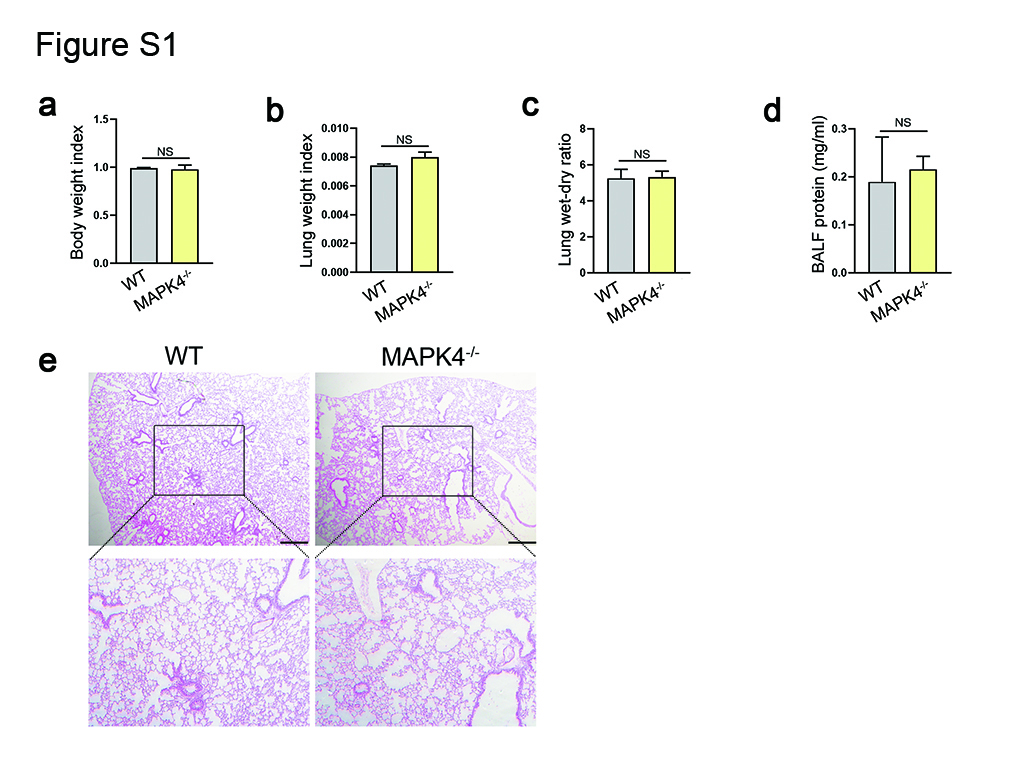
**

**Fig S1. The weight index and histology of lung tissues in MAPK4^-/-^ mice.**

WT C57BL/6 mice (n=6) and MAPK4^-/-^ mice (n=6) were sacrificed and the lung was collected. **a** The weight index, **b** lung weight index (lung weight/weight) and **c** lung wet-dry ratio were obtained, respectively. **d** The protein concentration in BALF was also detected. **e** The histology of lung tissues was analyzed by HE staining, respectively. Scale bar=50μm. Data were presented as the mean ± SEM. NS, no significance.

**
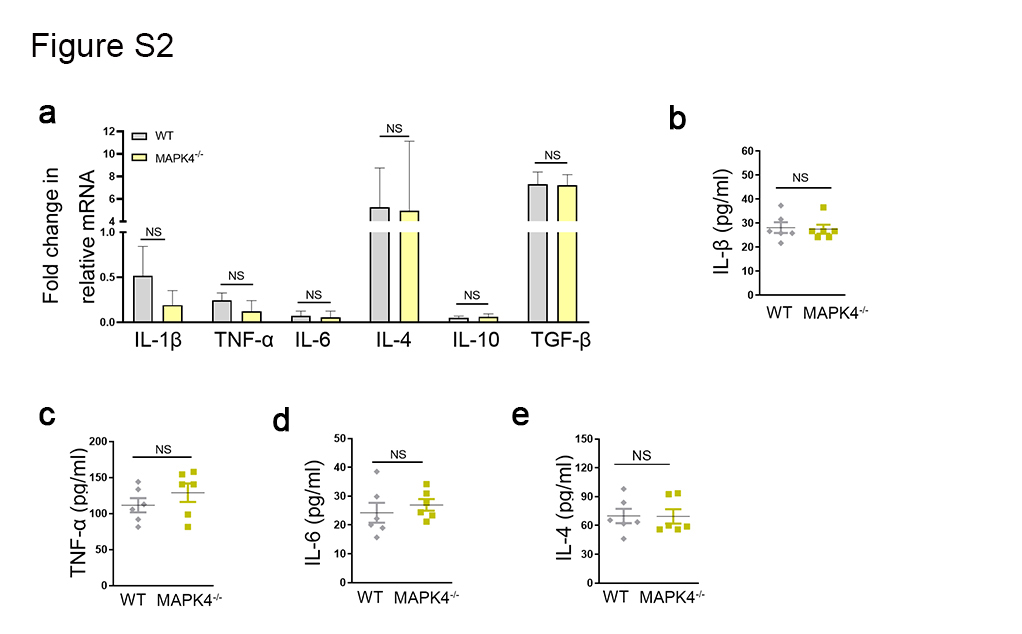
**

**Fig S2. The levels of related inflammatory cytokines in lung tissues of MAPK4^-/-^ mice.**

WT C57BL/6 mice (n=6) and MAPK4^-/-^ mice (n=6) were sacrificed and the lung was collected. **a** The mRNA levels of IL-1β, TNF-α, IL-6, TGF-β, IL-4 and IL-10 in lung tissues were detected by Real-time PCR assay and calculated. **b-e** The protein levels of IL-1β, TNF-α, IL-6 and IL-4 in BALF were detected by ELISA assay. Data were presented as the mean ± SEM. NS, no significance.

**
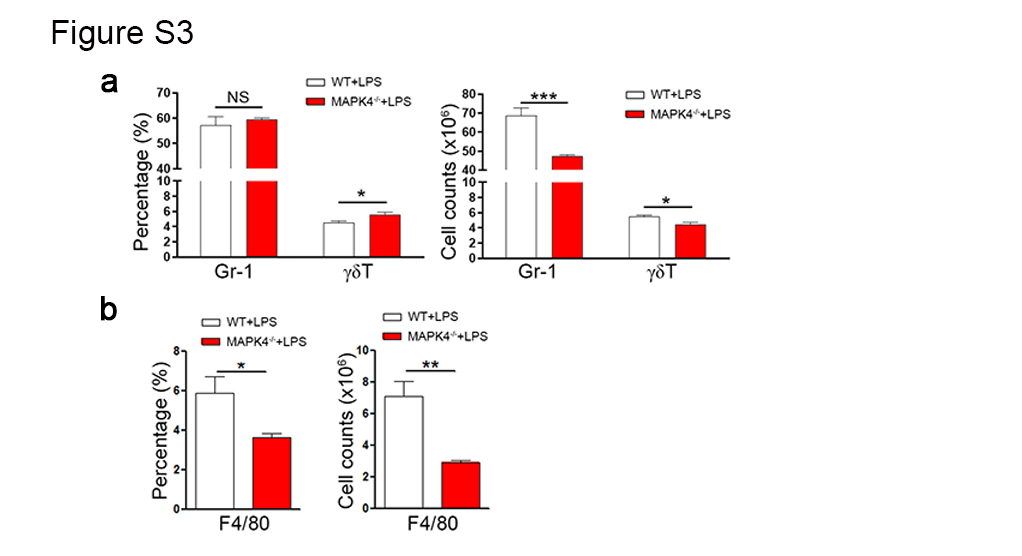
**

**Fig S3. MAPK4 deficiency alters the immune cells composition in spleen of ALI mice.**

WT C57BL/6 mice (n=6) and MAPK4^-/-^ mice (n=6) were administered with i.p. 10mg/kg LPS, 24h, respectively. Then, the spleen cells were collected. **a** The proportions of Gr-1^+^ neutrophils and γδT^+^ cells were analyzed by FCM and the absolute numbers of these cells were calculated, respectively. **b** The proportion of F4/80^+^ Mφ was analyzed by FCM and the absolute number of Mφ was calculated, respectively. Data were presented as the mean ± SEM. *p<0.05, **p<0.01, ****p*<0.001.

**
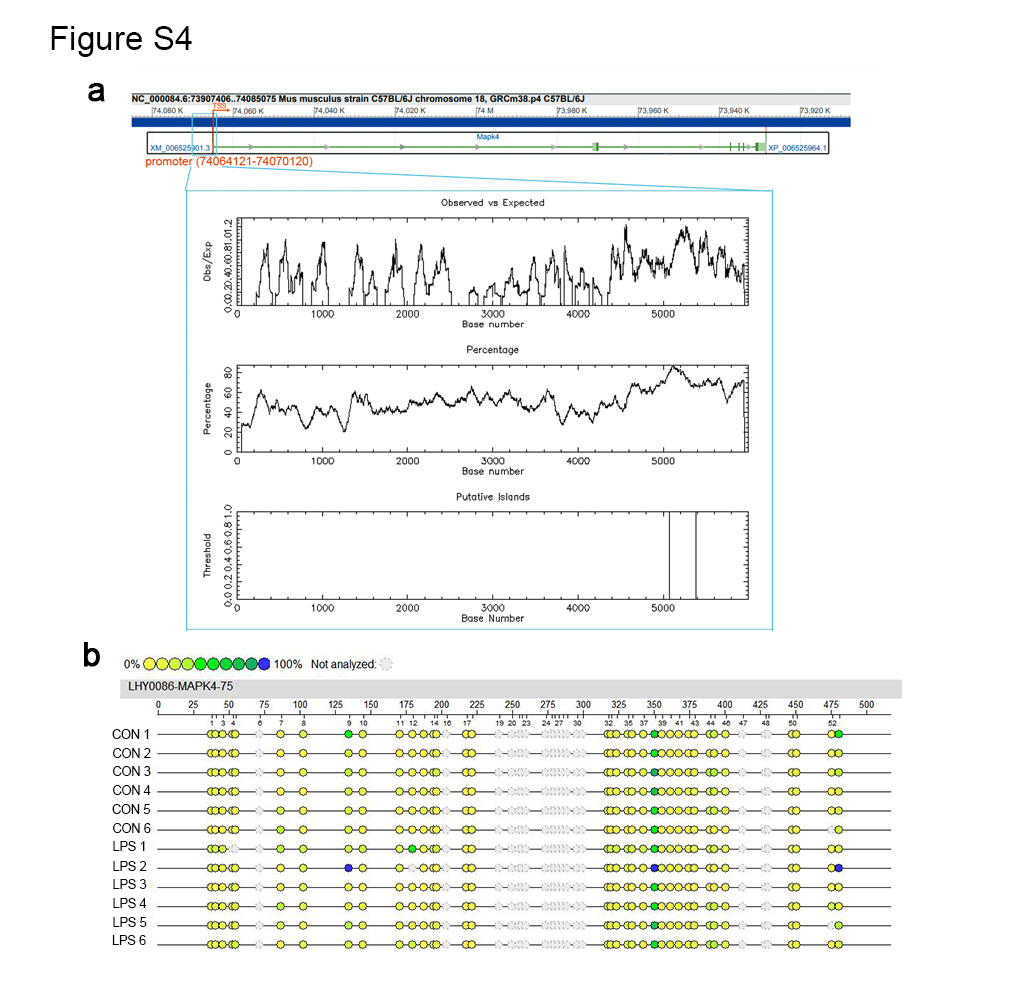
**

**Fig S4. CpG methylation levels of MAPK4 promoter in ALI mice.**

WT C57BL/6mice (n=6) were administered with i.p. 10mg/kg LPS or PBS, respectively. After 24h, lung tissues were obtained and DNA was extracted. **a** The putative CpG island was found in the promoter of MAPK4 at *http://www.ebi.ac.uk/Tools/seqstats/emboss_cpgplot/*. **b** Methylation levels of CpG sites in MAPK4 promoter were analyzed by massARRAY assay.


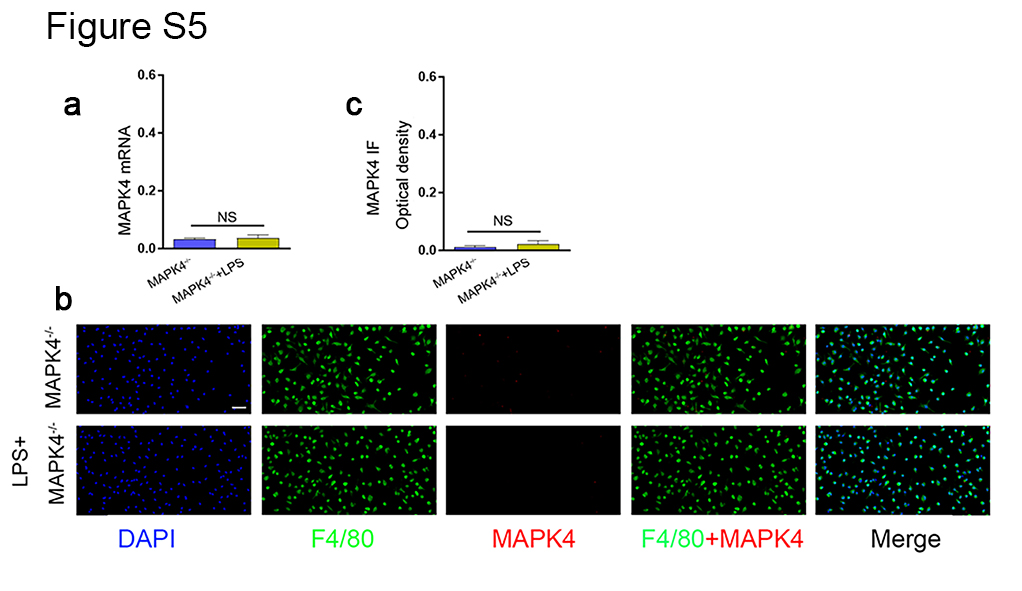


**Fig S5. The expression of MAPK4 in MAPK4 deficiency macrophages.**

**a** The mRNA levels of MAPK4 were detected by Real-time PCR assay in MAPK4^-/-^ macrophages and LPS-treated MAPK4^-/-^ macrophages (n=3). **b-c** The protein levels of MAPK4 in MAPK4^-/-^ macrophages and LPS-treated MAPK4^-/-^ macrophages were detected by immunofluorescence and calculated (n=3). Scale bar=25μm. Data were presented as the mean ± SEM. *p<0.05, **p<0.01.
